# Carbonation Curing on Magnetically Separated Steel Slag for the Preparation of Artificial Reefs

**DOI:** 10.3390/ma15062055

**Published:** 2022-03-10

**Authors:** Jiajie Li, Shaowei Zhao, Xiaoqian Song, Wen Ni, Shilong Mao, Huihui Du, Sitao Zhu, Fuxing Jiang, Hui Zeng, Xuejie Deng, Michael Hitch

**Affiliations:** 1 Key Laboratory of Ministry of Education for Efficient Mining and Safety of Metal Mines, School of Civil and Resource Engineering, University of Science and Technology Beijing, No. 30 Xueyuan Road, Haidian District, Beijing 100083, China; jiajieli@ustb.edu.cn (J.L.); zhaoshaowei5138@163.com (S.Z.); maoshil@126.com (S.M.); huihuidu@xs.ustb.edu.cn (H.D.); jiangfuxing1@163.com (F.J.); 2 State Key Laboratory of Coal Resources and Safe Mining, China University of Mining and Technology, No. 1 University Road, Xuzhou 221116, China; 3 Shandong Iron and Steel Group Co., Ltd., No. 2000 Shunhua Road, Gaoxi District, Jinan 250101, China; zh13863458091@163.com; 4 China Institute of Urban Governance, Shanghai Jiao Tong University, Shanghai 200030, China; 5 School of International and Public Affairs, Shanghai Jiao Tong University, Shanghai 200030, China; 6 School of Energy and Mining Engineering, China University of Mining and Technology, Beijing 100083, China; dengxj@cumtb.edu.cn; 7 Western Australian School of Mines: Minerals, Energy and Chemical Engineering, Curtin University, P.O. Box U1987, Perth, WA 6845, Australia

**Keywords:** carbonation curing, steel slag powder, steel slag mud, seawater curing, artificial reefs

## Abstract

Magnetic separation is an effective method to recover iron from steel slag. However, the ultra-fine tailings generated from steel slag become a new issue for utilization. The dry separation processes generates steel slag powder, which has hydration activity and can be used as cement filler. However, wet separation processes produce steel slag mud, which has lost its hydration activity and is no longer suitable to be used as a cement filler. This study investigates the potential of magnetically separated steel slag for carbonation curing and the potential use of the carbonated products as an artificial reef. Steel slag powder and steel slag mud were moulded, carbonation-cured and seawater-cured. Various testing methods were used to characterize the macro and micro properties of the materials. The results obtained show that carbonation and hydration collaborated during the carbonation curing process of steel slag powder, while only carbonation happened during the carbonation curing process of steel slag mud. The seawater-curing process of carbonated steel slag powder compact had three stages: C-S-H gel formation, C-S-H gel decomposition and equilibrium, which were in correspondence to the compressive strength of compact increasing, decreasing and unchanged. However, the seawater-curing process of carbonated steel slag mud compact suffered three stages: C-S-H gel decomposition, calcite transfer to vaterite and equilibrium, which made the compressive strength of compact decreased, increased and unchanged. Carbonated steel slags tailings after magnetic separation underwent their lowest compressive strength when seawater-cured for 7 days. The amount of CaO in the carbonation active minerals in the steel slag determined the carbonation consolidation ability of steel slag and durability of the carbonated steel slag compacts. This paper provides a reference for preparation of artificial reefs and marine coagulation materials by the carbonation curing of steel slag.

## 1. Introduction

Steel slag is produced in the steelmaking process. China produced 160 Mt of steel slag in 2020. However, the actual utilization rate of steel slag is only 30% [1]. The stockpiling of abandoned steel slag occupies a great deal of land, pollutes the environment and wastes resources. The Chinese government revised the Environmental Protection Law in 2018 and charged an environmental protection tax for the stockpiling of smelting slag. Iron and steel industries have to ensure that they stabilize and utilize their slags.

Researchers have focused on the study of application of the steel slag in various ways, including aggregates in road construction, fillers of cementitious materials, as well as iron recycling [2]. Steel slag has great advantages in its use as aggregates, due to its high mechanical performance and wear resistance [3]. Howev er, using steel slag as aggregates does not embody the high value of steel slag. Steel slag contains hydraulic materials (i.e., C_2_S and C_3_S), which could replace part of the cementing materials [4,5,6]. 

However, the hydration activity of steel slag is too low to consolidate at the initial curing age [7,8]. In addition, the free oxides (f-CaO, f-MgO) in steel slag would lead to volume expansion and insatiability [9]. Magnetic separation can recover iron from the steel slag. However, the tailings (i.e., steel slag powder and steel slag mud) after magnetic separation become a new problem. Searching for proper strategies for the use of steel slag tailings after iron recycling would assist with high-value utilization of steel slag with a near 100% utilization rate.

Steel slag tailings after iron recycling contain a large amount of calcium oxide, calcium hydroxide, calcium silicates, calcium aluminates and calcium ferrates, which are suitable for mineral carbonation [10,11,12,13]. At present, mineral carbonation on steel slag has been investigated in two aspects: indirect carbonation to generate pure CaCO_3_ [14] and direct carbonation to produce construction materials [15]. Between both aspects investigated, the direct carbonation process on steel slag for building material preparation is similar to the traditional hydration curing process, which is simple and involves low energy consumption [16]. 

The carbonated steel slag materials have a high value of early compressive strength, which reaches 30–100 MPa after being carbonation-cured for 1 day [17,18]. The carbonated steel slag materials are environmentally clean, have a neutral pH [19] and are capable of consolidating heavy metals [20]. The carbonated steel slag materials also have good durability qualities, such as volume stability, frost resistance, CO_2_ corrosion resistance and ion penetration resistance [21,22]. 

At present, a large number of experts have studied the effects of carbonation curing on the performances of carbonated steel slag materials [16,23], including the curing time [24,25], curing temperature [26], CO_2_ pressure [27] and mineral composition of steel slag [28,29]. However, only a few studies have been focused on the carbonation curing of steel slag tailings, especially on the wet-ground steel slag.

Carbonated steel slag materials are mainly composed of calcium carbonates, which is the components of shell. Thus, carbonated steel slag materials are extremely suitable when used as an artificial reef, which provides a place for marine organisms to live and breed [30]. Carbonation curing of concrete artificial reefs has recognized steel slag materials as a good strategy to reduce the surface pH, which enhances their attraction to marine organisms [31,32]. JFE Steel Corporation developed a carbonated steel slag artificial reef (Marine Block ^TM^), which maintained good stability after 5 years of application [33,34]. However, the durability and mechanism of carbonated steel slag tailings in seawater has not been studied.

This study investigated the mechanism of carbonation curing on steel slag tailings after iron recycling and the potential application of carbonated steel slag tailings as artificial reefs. Laboratory experiments were performed to study the changes of compressive strength and carbonation degree of carbonated steel slag powder and steel slag mud under carbonation curing and seawater curing. 

Various characterization methods were used to study the microstructure changes of carbonated steel slag tailings to reveal their strength evolution mechanism. The results of this study provide a reference for the utilization of steel slag tailings after iron recycling and the development of carbonated artificial reef concrete.

## 2. Materials and Methods

### 2.1. Materials

Pristine steel slag was provided by Shandong Iron and Steel Co., Ltd. (Jinan, Shandong, China). Steel slag was ground in an SMΦ 500 × 500 mm^2^ ball mill for 30 min and was screened using a 60 mesh Taylor standard sieve. The under size were collected and chosen as the steel slag powder (SSD) in the experiments. The density of SSD was 3.4 g/cm^3^ and the Blaine’s number of SSD was 379 m^2^/kg.

Pristine steel slag mud was provided by Handan Iron and Steel Co., Ltd. (Handan, Hebei, China). The congealed steel slag mud was ground and screened using the same method as SSD. The obtained steel slag mud (SSW) had a density of 3.3 g/cm^3^ and a Blaine’s number of 335 m^2^/kg.

Table 1 lists the oxide compositions of SSD and SSW, which were tested by X-ray fluorescence (XRF) spectroscopy, (XRF-1800, Shimadzu, Kyoto, Japan). Alkalinity (*A*) is the value of the mass fraction of CaO over the mass fraction of P_2_O_5_ and SiO_2_ (Equation (1)) [35], which represents the activity of the steel slag. According to Equation (1), the alkalinity of SSD and SSW were 2.5 and 2, respectively, which fall in the range of medium alkalinity.

(1) $$A=\frac{m_{\mathrm{CaO}}}{m_{{\mathrm{SiO}}_{2}}+m_{P_{2}O_{5}}},$$

where $m_{\mathrm{CaO}}$ is the mass fraction of CaO in steel slag, $m_{P_{2}O_{5}}$ is the mass fraction of P_2_O_5_ in steel slag, and $m_{{\mathrm{SiO}}_{2}}$ is the mass fraction of SiO_2_ in steel slag as tested by XRF.

**Figure 1:**
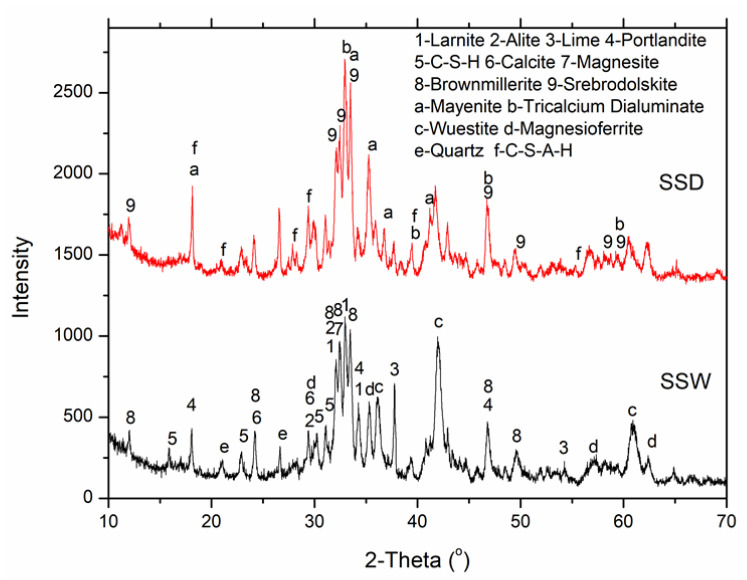
XRD of the steel slag powder (SSD) and steel slag mud (SSW).

The mineral composition of SSD and SSW were tested by quantitative X-ray diffraction (QXRD), and the results are shown in Table 1 and Figure 1. The SSD and SSW contained similar phases, including calcium silicate salts (larnite (C_2_S) and alite (C_3_S)), calcium ferrates or/and aluminates (srebrodolskite (C_2_F), brownmillerite (C_2_FA), mayenite (C_12_A_7_) and dialuminate (C_3_A)), oxides (lime (CaO), quartz (SiO_2_), RO phase (FeO, MgO and MnO) and hydrates (portlandite (Ca(OH)_2_) and clinoptilolite (C-S-H)). However, the mineral compositions of SSD and SSW are different. SSW contained a greater RO phase but a lesser quantity of calcium ferrates or/and aluminates compared with SSD.

### 2.2. Experimental Method

**Figure 2:**
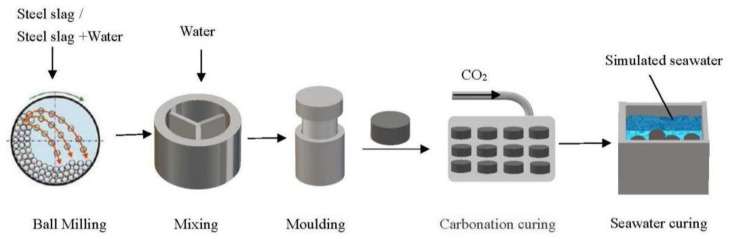
The flow chart of the experiments.

Figure 2 shows the flow chart of the experiments. SSD or SSW was mixed with water, moulded, carbonation-cured and seawater-cured. The samples before and after carbonation curing and seawater curing were characterized by various methods.

#### 2.2.1. Mixing and Moulding

Tap water and steel slag tailings were mixed at a ratio of 1.5:10 in a paste mixer for 2 min. We placed 8 g of the moisture material into a 20-mm diameter cylindrical compression stainless steel moulded and compacted into individual specimens at a uniaxial load of 9 MPa for 1 min before demoulded.

#### 2.2.2. Carbonization Curing

The demoulded specimens were placed in a carbonation chamber (CABR-HTX12, China Academy of Building Research, Beijing, China) for curing. Carbonation curing conditions were maintained at a temperature of 20 ± 3 °C, relative humidity of 70 ± 2% and CO_2_ concentration of 20 ± 3 vol %. The specimens after carbonization curing for 1, 3, 6, 12 and 24 h were taken out for seawater curing and material characterization.

#### 2.2.3. Seawater Curing

The simulated seawater was prepared according to ASTM D1141-1998(2008) Standard Practice for the Preparation of Substitute Ocean Water. Table 2 lists the dosage of each ingredient of simulated seawater. The simulated seawater was placed in a container, where the compacts before and after carbonation could be immersed for curing. Seawater curing was conducted at a temperature of 20 ± 1 °C. The compacts after seawater curing for 3, 7, 14, 28 and 56 days were collected for material characterization.

#### 2.2.4. Material Characterization

Uniaxial compressive strength was measured in a digital pressure testing machine (YES-300, Jinan Chenda Test Machine Manufacturing Co., Ltd., Jinan, China). In each test, the compact was placed in the center of the workbench and pressure was exerted at a speed of 0.05 kN/s.

The carbon contents of the samples were measured using a carbon/sulphur combustion analyser (EMIA-820 V, Horiba, Kyoto, Japan). In each test, 0.2–0.3 g samples were placed into a combustion crucible and covered with 1 g flux (90% tungsten, 10% tin, C < 0.0008%). The carbon content of the sample was determined after combustion up to 1050 °C.

The mineralogy of the carbonated compacts was characterized using the Quantitative X-ray diffraction (QXRD) method. The X-ray diffraction patterns of the samples were measured using an X-ray diffractometer (D/Max-RB, Rigaku, Tokyo, Japan) equipped with a Cu-Kα radiation (20 kV, 10 mA) source, working in 2θ geometry with a recorded range from 3 to 70°; with a step size of 0.02° in the step-scanning mode (FT 0.7 s). 

A pattern of standard sample Si (SRM 640c) was collected using the same procedure and was used to obtain the instrumentally broadened profile, as suggested by the US National Institute of Standards and Technology (NIST). The X-ray diffractograms were analysed using the International Centre for Diffraction Data-base (ICDD) PDF-4 and Search-Match software X’Pert HighScore Plus version 3.0 (PANalytical B.V., Almelo, Netherlands). The X-ray powder diffraction data of the samples were refined using the Rietveld method for quantitative analysis. All the QXRD results were obtained when the weighted-profile R value was below 10%.

Thermogravimetric differential thermal analysis (TG-DTA) was tested using a TG-DTA (thermogravimetry and differential thermal analysis) analyser (STA 449F3, Netsch, Selb, Germany). The tests were performed under an argon atmosphere with a flow rate of 20 mL/min. The heating rate was 10 °C/min, and the temperature range was 50 to 1000 °C.

#### 2.2.5. CO_2_ Uptake Calculation

The carbon content ($m_{C}$) of the sample was converted to CO_2_ uptake capacity ($m_{{\mathrm{co}}_{2}}$) using Equation (2).

(2) $$m_{{\mathrm{co}}_{2}}=\frac{m_{C}}{MW_{C}}\times MW_{{\mathrm{CO}}_{2}},$$

where the $MW_{C}$ is the molar mass of C is 12 g/mol, and $MW_{{\mathrm{CO}}_{2}}$ is the molar mass of CO_2_ is 44 g/mol.

Assuming that only the mineral containing CaO takes part in the carbonation during the carbonation curing on steel slag. Although minerals containing MgO and Fe_2_O_3_ could also react with CO_2_, the reaction rate is extremely slow under the carbonation-curing conditions adopted in this study and can be ignored during the calculations. The CO_2_ uptake or carbonation conversion ($R_{x}$) of steel slag can be calculated according to Equation (3):

(3) $$R_{x}=\frac{\frac{m_{{\mathrm{co}}_{2}}}{1-m_{{\mathrm{co}}_{2}}}\times \frac{MW_{\mathrm{CaO}}}{MW_{{\mathrm{CO}}_{2}}}}{M_{\mathrm{CaO}}},$$

where, $MW_{\mathrm{CaO}}$ is the molar mass of CaO, which is 56 g/mol, and $M_{\mathrm{CaO}}$ is the mass fraction of CaO in the steel slag. The carbon content of SSD and SSW were 1.37% and 0.64%, respectively. According to Equations (2) and (3), the carbonation conversion of SSD and SSW were 16.7% and 7.6%, respectively.

All calculated data are the average value from three tests with the standard deviation.

## 3. Results and Discussion

### 3.1. The Effect of Carbonation Time on the Compressive Strength and CO_2_ Uptake of Compacts

#### 3.1.1. The Changes in Carbonation Conversion

**Figure 3:**
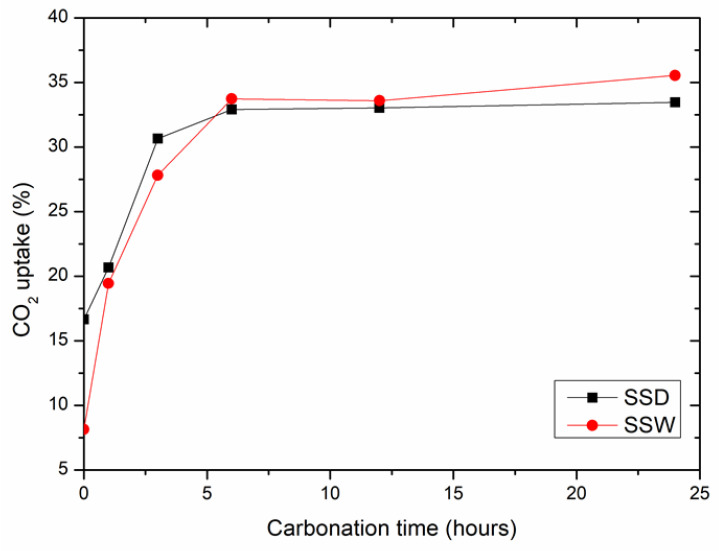
The effect of carbonation time on CO_2_ uptake of compacts made from the SSD and SSW. The standard deviations of CO_2_ uptake ranged from 0.26% to 1.28%.

Figure 3 shows the CO_2_ uptake of SSD and SSW compacts after carbonation curing for various durations. The CO_2_ uptake of the compacts increased quickly during the first 6 h of carbonation curing. Afterwards, it became extremely slow or nearly unchanged. The result is consistent with Mo et al. [36], who found that carbonation curing formed a densely arranged CaCO_3_ crystal layer on the surface of the compact, which blocked CO_2_ from entering into the core and prevented further reaction.

The CO_2_ uptake rates of SSD and SSW before carbonation curing were 16.7% and 7.6%, respectively. This indicates that both SSD and SSD were carbonated at atmospheric conditions. The CO_2_ uptake of SSD was higher than that of SSW. The CO_2_ uptake of SSD and SSW compacts after carbonation curing for 24 h were 33.5% and 35.5%, respectively. The correspondence to increases in CO_2_ uptake of SSD and SSW compact during carbonation curing for 24 h were 16.8% and 27.9%, respectively. The carbonation rate of the SSW was higher than that of SSD under the same carbonation curing conditions.

#### 3.1.2. The Changes in Compressive Strength

**Figure 4:**
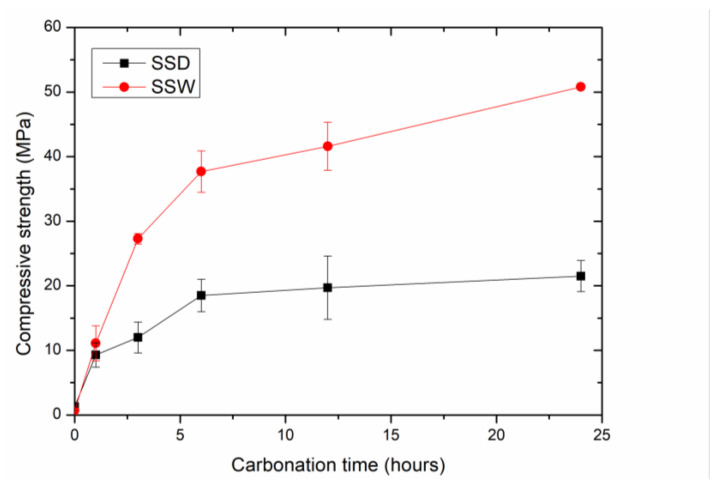
The effect of carbonation time on the compressive strength of the compacts made from SSD and SSW.

Figure 4 shows the compressive strength of SSD and SSW compacts after carbonation curing for various times. Similar to the CO_2_ uptake, the compressive strength of compacts increased quickly during the first 6 h and then slowed down. The compressive strength of SSD and SSW compacts before carbonation curing were 1.3 and 0.7 MPa, respectively. The higher compressive strength of SSD than SSW may be due to hydration during the mixing and moulding, which contributes to consolidation of the compacts. However, SSW had little hydration reactivity, which was consumed during the wet grinding.

The compressive strengths of the carbonated SSW compacts were higher than that of carbonated SSD compacts at all the stages of carbonation curing. After carbonation curing for 24 h, the compressive strengths of carbonated SSW compacts and carbonated SSD compacts were 50 MPa and 21 MPa, respectively. The results of compressive strength are similar to that of CO_2_ uptake. This indicates that carbonation (other than hydration) is the main reason for the consolidation of SSD and SSW compacts during carbonation curing.

#### 3.1.3. Relationship between the Compressive Strength and Carbonation Conversion

**Figure 5:**
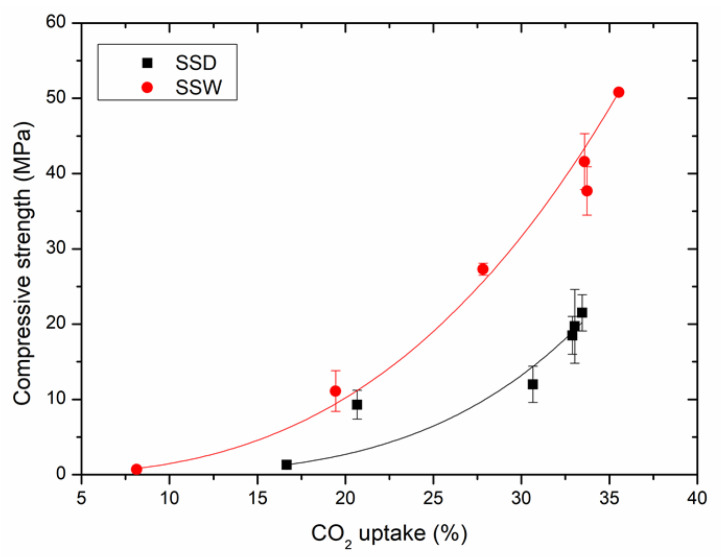
The relationship between the compressive strength and carbonation degree of SSD and SSW.

Figure 5 shows the relationship between the compressive strength and CO_2_ uptake of SSD and SSW compacts. The compressive strength of carbonated SSW and SSD compacts are exponential to their CO_2_ uptake, as shown in Equations (4) and (5), which are the fitting equations of the data from Figure 5. The result is in line with Wang et al. [17] who found that the compressive strength of steel slag is linear to the carbonation conversion. As shown in Figure 5, the compressive strength of SSW is higher than that of SSD with the same CO_2_ uptake. This indicates that the carbonation consolidation efficiency of SSW is higher than that of SSD. Material characterizations are necessary to find out the reason for the changes.

SSW: *y* = 0.002 · *x*^2.79^ *R*^2^ = 0.99 (4)

SSD: *y* = 2 × 10^−5^· *x* ^3.91^ *R*^2^ = 0.91 (5)

### 3.2. The Effect of Seawater Curing Time on the Compressive Strength of Carbonated Compacts

**Figure 6:**
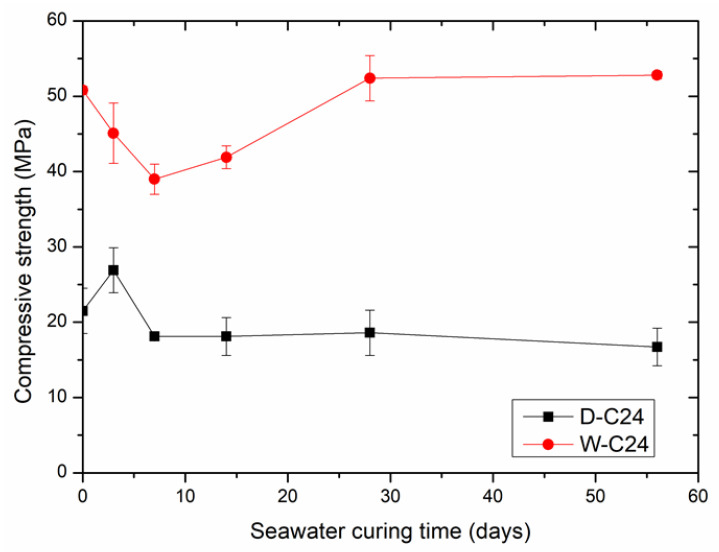
The effects of seawater curing time on the compressive strength of carbonated compacts made from SSD (D-C24) and SSW (W-C24).

To investigate the feasibility of carbonated steel slag compacts used as an artificial reef, the 24 h carbonation-cured SSD compacts (D-C24) and SSW compacts (W-C24) were placed inside the artificial seawater at room temperature and cured up to 56 days. Figure 6 shows the changes in the compressive strength of D-C24 and W-C24 with seawater curing time. The compressive strength of D-C24 and W-C24 were higher than 18 MPa at any of the stages of seawater curing, which meets the requirement of an artificial reef (no less than C20) [37].

As shown in Figure 6, the compressive strength of D-C24 increased from 21.5 to 27 MPa after the seawater curing for 3 days. Then, it decreased dramatically to 18 MPa after seawater curing for 7 days. Afterwards, it maintained the same with further seawater curing. The increase in compressive strength of D-C24 in the early stage of seawater curing may be due to hydration of the remaining C_2_S and/or C_3_S in the compact, which generated a large number of hydration products (i.e., C-S-H gel) with the appearance of enough water, and this hardened the compact. The decrease in the compressive strength of SSD-C24 in the late stage of seawater curing may be due to corrosion of the newly formed hydration products by the ions in the simulated seawater (i.e., Na^+^, K^+^, Mg^2+^, Ca^2+^, Cl^−^, Br^−^, HCO_3_^−^, etc.).

The compressive strength of W-C24 decreased from 50 to 39 MPa after the seawater curing for 7 days. Then, it increased back to the initial level after seawater curing for 28 days. Afterwards, it maintained the same with further seawater curing. Different from D-C24, W-C24 did not experience an increase in compressive strength at the early stage of seawater curing. This is mainly due to the fact that SSW had little hydration activity, which was consumed during the wet grinding. The rebound in the compressive strength of W-C24 in the middle stage of seawater curing, may be due to the generation of a new phase or the improvement of the microstructure of W-C24. Material characterization, such as QXRD, TGA and SEM are needed to find out the reason.

Compared to the comprehensive strength of compacts before the seawater curing, the compressive strength of D-C24 decreased by 30% after 56 days of seawater curing, while the compressive strength of W-C24 was nearly the same after 56 days of seawater curing. The carbonated SSW compacts may have a stronger resistance to seawater erosion and are more suitable as artificial reefs than carbonated SSD compacts.

Both D-24 and W-24 had their lowest compressive strength after seawater curing for 7 days. Therefore, 7 day of seawater curing of carbonated steel slag artificial reefs before application is preferable to confirm their reliability and durability.

### 3.3. QXRD Analysis

**Figure 7:**
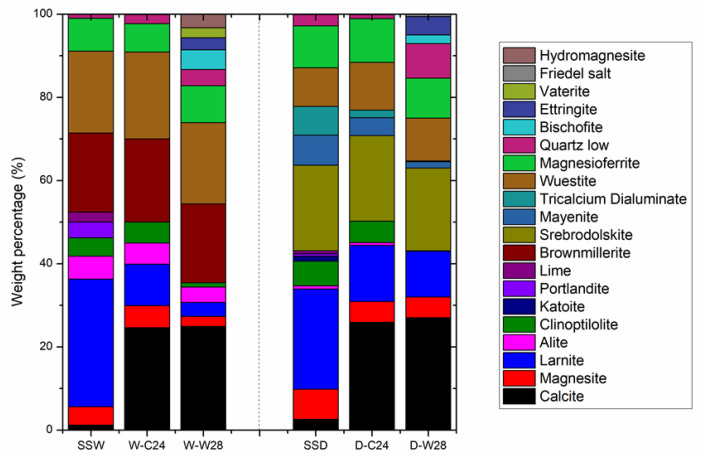
QXRD Analysis on the representative samples.

The QXRD analysis was performed to study changes in mineral composition occurring in the SSD and SSW compacts during the carbonation curing and seawater curing. Figure 7 shows the results of QXRD on the initial steel slag tailings (SSD and SSW), carbonated compacts (D-C24 and W-C24) and the carbonated compacts seawater-cured for 28 days (D-W28 and W-W28).

As shown in Figure 7, the CaCO_3_ substantially increased and MgCO_3_ slightly decreased in the compacts after 24 h of carbonation curing. This indicates that calcium-containing minerals are the main minerals being carbonated during the carbonation curing at room temperature. After the carbonation curing, the calcium oxide, hydroxide and hydration minerals (CaO, Ca(OH)_2_, C-S-H and C-S(A)-H) disappeared. 

The quantity of calcium silicates (C_2_S, C_3_S) and calcium aluminates (C_12_A_7_ and C_3_A) decreased significantly. While the quantity of calcium ferrates (C_2_F and C_2_FA) remained unchanged. This indicates that calcium oxide, hydroxide, hydration minerals, silicates and aluminates are carbonation active minerals, while calcium ferrates are carbonation inert minerals. The order of carbonation activities from high to low are calcium oxide, hydroxide and hydration minerals > calcium silicate and aluminates > calcium ferrates.

The weight percentage of CaO in SSW was lower than that in SSD. Correspondingly, the alkalinity of the SSW was lower than that of SSD (Table 1 and Figure 7). However, the CO_2_ uptake of SSW during carbonation curing is much higher than that of SSD. This indicates that the mineral compositions, other than weight percentage of CaO, are the key factors that control the CO_2_ uptake of steel slag during carbonation curing. The total weight percentages of calcium oxide, hydroxide and hydration minerals in SSW and SSD were 11.7% and 8.5%, respectively. 

The high amount of extremely reactive CaO may be the main reason for the high CO_2_ uptake of SSW in the early carbonation-curing stage (as shown in Figure 7). The weight percentage of calcium silicates in SSW and SSD were 36.2% and 25%, respectively, and the weight percentage of calcium aluminates in SSW and SSD were 0% and 14.1%, respectively. The total weight percentage of CaO in the silicates and aluminates of SSW and SSD were 24.7% and 16.2%, respectively. This indicates that a higher weight percentage of CaO in carbonation active minerals in SSW is the main reason for its higher CO_2_ uptake than that of SSD.

The weight percentage of calcium ferrates in the SSW and SSD were 19% and 20.6%, respectively. Due to the inert carbonation activity and a comparable quantity in SSD and SSW, the influence of calcium ferrates on the carbonation conversion of steel slag tailings is omitted in this study. However, Wang and Chang [38] found that carbonation of calcium ferrates generated carbonates with a large number of defects, which had an adverse effect on the strength development of steel slags during carbonation curing. Reduction of calcium ferrates before carbonation curing would be a good strategy to enhance the quality of carbonated steel slag materials.

The weight percentage of Fe_2_O_3_ tested by XRF in SSW and SSD were 27.75% and 24.48%, respectively. Although the weight percentage of calcium ferrates in both samples were similar, the weight percentage of RO phase (FeO and (Mg, Fe)_2_O_3_) in SSW (27.6%) was significantly higher than that in SSD (19.4%). This indicates that RO phase had little impact on the CO_2_ uptake and compressive strength of SSW during carbonation curing, due to the extremely low carbonation activity of RO phase at room temperature. The results are in line with Chen et al. [39], who found that the carbonation of RO needed to be activated under high temperature.

The changes in mineral composition of the carbonated SSD and SSW compacts after the seawater curing for 28 days were similar. The amount of calcium carbonates remained unchanged, which indicates that the carbonation process was nearly terminated during seawater curing. Calcium silicates and calcium aluminates were continuously being consumed and formed a series of hydration products (i.e., ettringite, Friedel salt, hydromagnesite and bischofite), due to the hydration of carbonated compacts with the presents of Cl^−^, SO^2−^ and Mg^2+^. The amount of hydromagnesite and bischofite in W-W28 was higher than that in D-W28, which is mainly due to the high Mg content in SSW. The amount of ettringite and Friedel salt in D-W28 was higher than that in W-W28, which is due to the high Al content in SSD. 

In addition, SiO_2_ was generated in both D-C24 and W-C24 after 28 days of simulated seawater curing. The generation of SiO_2_ may be due to the decomposition of C-S-H gel in the seawater. The amount of SiO_2_ in D-W28 was higher than that in W-W28. This indicates that the newly formed C-S-H gel in D-C24 is quite easily being decomposed in the seawater environment. The results of QXRD are in line with that of the compressive strength.

According to the observation above, the following hypophysis can be addressed. The increase in the compressive strength of D-C24 after seawater curing for 3 days, is due to the generation of a large amount of C-S-H gels. The reduced compressive strength of D-C24 and W-C24 after the seawater curing up to 7 days are due to the decomposition of C-S-H gels. 

The strength of D-C24 remained unchanged after seawater curing for 7 days when the rate of generation of hydration products (ettringite, Friedel salt and hydromagnesite), and the rate of C-S-H gel decomposition reached equilibrium. The strength of W-C24 continued to increase after the seawater curing for 7 days, which may be due to the formation of hydromagnesite and vaterite. TG-DTG and SEM-EDS tests are necessary to confirm the findings.

### 3.4. TG-DTG Analysis

**Figure 8:**
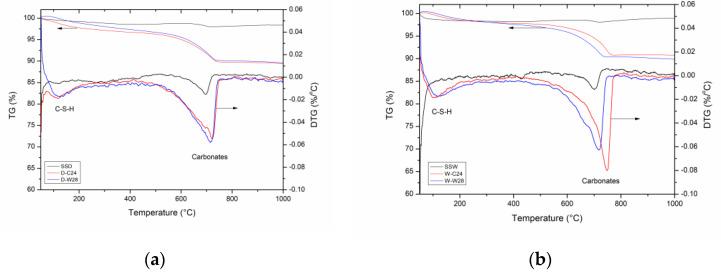
TG-DTA analysis of (**a**) SSD and (**b**) SSW.

The hydration and carbonation products of SSD could be effectively analysed by differential thermal analysis. Figure 8 shows the TG-DTG curves for the SSD and SSW and their carbonated products and seawater-cured products.

The intermolecular water and hydration products thermogravimetry occurred at <240 °C and 240–500 °C. Among them, 60–200 °C is the weight loss of C-S-(A)-H gel and ettringite, 200–400 °C is the dehydration of monocarboaluminate (C_3_A·CaCO_3_·11H_2_O) [40], while 450 °C is the weight loss of OH^-^ in Ca(OH)_2_. The decomposition of carbonates (i.e., calcite, aragonite, monocarboaluminate) was at 500–800 °C [41].

As shown in Figure 8, SSD and SSW already contained some carbonate. The result is consistent with the result of total carbon. The carbonate in the SSD and SSW compacts increased significantly after carbonation curing for 24 h. The decomposition temperatures of the carbonates in D-C24 and W-C24 were 710 and 750 °C, respectively. This indicates that the crystalline carbonates generated from carbonation curing of SSW were more stable than that of SSD. 

Morandeau et al. [42] found that aragonite and vaterite had low decomposition temperatures, while the well-crystallized calcite was in the range of 750–900 °C decomposition. This indicates that the aragonite and vaterite are the main carbonates in D-C24, while calcite is the main carbonate in W-C24. The result is not in line with Chang et al. [43], who studied the effect of Ca-Si ratio on the carbonation curing of C-S-H gel. 

They found that the large proportion of aragonite and vaterite were formed at low Ca-Si ratios, while calcite was formed at high Ca-Si ratios. This is mainly due to the variation in raw material being carbonation-cured. In the case of carbonation curing on a single phase that has the same carbonation activity, Ca-Si ratios could control the crystal structure of carbonation products. Steel slag contains various types of CaO containing minerals, which have different carbonation activities. The quantity of calcium oxide in carbonation active minerals may be the main reason for the formation of different carbonated products after carbonation curing. A large amount of calcium oxide in carbonation active minerals (SSW) is preferred to produce calcite after the carbonation curing. While a low amount of calcium oxide in carbonation active minerals (SSD) tend to form aragonite and vaterite after carbonation curing.

Figure 8 shows that the carbonate phase in both D-C24 and W-C24 transformed to the phase that could decompose at a lower temperature. The carbonation phase transfer in W-C24 is clearer than that in D-C24. This indicates that seawater curing could turn the carbonation phase from a thermo-stable structure to a relatively thermo-unstable structure.

Table 3 lists the weight loss percentage of six samples in defined temperature intervals calculated according to TG results in Figure 8. In the range of 50–240 °C, the weight loss percentages of SSD and SSW were 0.9% and 1.6%, respectively. This indicates that SSW contains a higher amount of C-S-H gels and ettringite compared with SSD. The hydration products in SSW is formed during the wet grinding. In the range of 240–500 °C, the weight loss percentages of SSD and SSW were 0.5% and 0.2%, respectively. Since Ca(OH)_2_ in SSW was higher than that in SSD, as shown in the QXRD results, SSD may contain a larger amount of monocarboaluminate compared with SSW. In the range of 500–800 °C, the weight loss percentage of SSD and SSW were 0.5% and 0.3%, respectively. This indicates that both SSD and SSW were already being carbonated with CO_2_ in the air.

After the carbonation curing for 24 h, the amount of monocarboaluminate increased slightly (240–500 °C), and the amount of carbonates (500–800 °C) increased dramatically in both SSD and SSW compacts. The amount of C-S-H gels and ettringite (50–240 °C) remained unchanged in the SSW compact, while it increased up to three times the original value in SSD compacts. This indicates that both hydration and carbonation occurred in SSD compacts, while only carbonation happened in SSW compacts during the carbonation curing process.

After seawater curing for 28 days, the amount of carbonates (500–800 °C) remained unchanged in both W-C24 and D-C24. The weight loss at 240–500 °C increased slightly, which correspond to the newly generated hydration products in W-W28 and D-W28 tested by QXRD. The amount of C-S-H gel and ettringite (50–240 °C) remained unchanged in W-C24 and decreased in D-C24. This indicates that the newly formed C-S-H gel in the SSD compacts during carbonation was decomposed during seawater curing.

### 3.5. SEM-EDS Analysis

**Figure 9:**
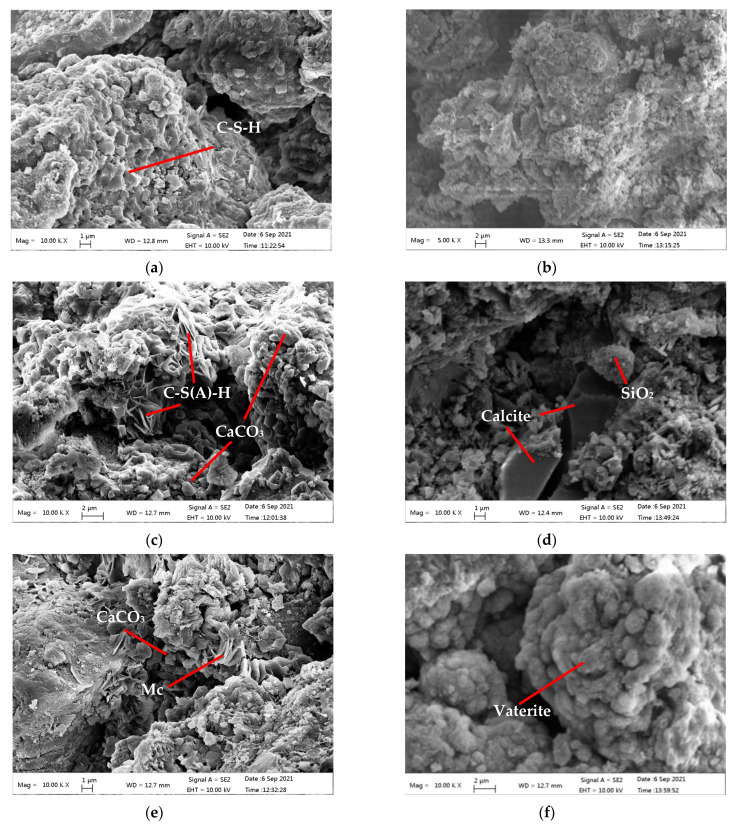

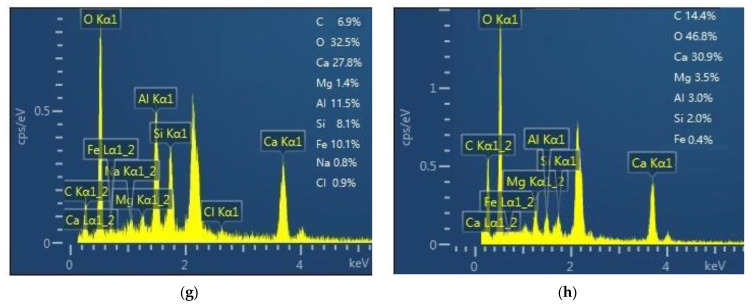
The morphology and EDS spectra of samples. (**a**) The morphology of SSD. (**b**) The morphology of SSW. (**c**) The morphology of D-C24. (**d**) The morphology of W-C24. (**e**) The morphology of D-W28. (**f**) The morphology of W-W28. (**g**) The EDS spectra of Mc in (**e**). (**h**) The EDS spectra of vaterite in (**f**). Mc denotes monocarboaluminate. % in EDS spectra are weight %.

Figure 9 shows the morphology of the SSD and SSW compacts before and after carbonation for 24 h (D-C24 and W-C24) and the carbonated compacts after seawater curing for 28 days (D-W28 and W-W28). The chemical composition of particles with typical morphology were tested by EDS spectra to assist with analysing their mineralogy [44,45,46]. As shown in Figure 9a,b, the particles in both SSD and SSW became aggregated and/or agglomerated after mixing and moulding. 

The aggregates in the SSD compacts were denser than those in the SSW compacts. This is consistent with results of compressive strength of SSD and SSW compacts before carbonation curing. The EDS results show that the C-S-H gel is the main cementing phase in the SSD compacts. This is in line with Wang et al. [29], who found that hydration was the main reason for the increase in strength in steel slag compacts during moulding. Thus, the loose structure of SSW compacts is due to the generation of little C-S-H gel during the mixing and moulding of SSW, whose hydration activity was reduced during wet grinding.

As shown in Figure 9 and the EDS results, D-C24 contains needle-like C-S(A)-H gel [40] and poorly crystallized CaCO_3_ [47]. While the W-C24 contains properly crystallized calcite and amorphous SiO_2_. This is in agreement with the XRD and TG-DTG results.

The carbonates in the D-W28 were composed of poor crystallized CaCO_3_ [47] and monocarboaluminate (C_3_A(F)·CaCO_3_·11H_2_O) [29]. This indicates that the continuous hydration of C_12_A_7_ and C_2_F in D-C24 occurred during the seawater curing. This is consistent with the results of XRD and TG-DTG.

Spherical vaterite [48] appears in W-W28. This is consistent with the result of TG-DTG. Vaterite seems more stable than calcite in the seawater. The elastic modulus of aragonite, calcite and vaterite are 5(4), 16(7) to 31(8) GPa, respectively [49]. Thus, the transformation of calcite to vaterite is the main reason for the increase in compressive strength of W-C24 after a long duration of seawater curing. It is important to note that only crystallized calcite was transformed to vaterite after seawater curing. Therefore, enhancing the calcite content in the carbonated steel slag compacts is necessary to improve their durability in the seawater. Measurements, such as increasing the carbonation active minerals in the steel slag, would assist in generation of calcite during carbonation curing.

## 4. Conclusions

This paper reports the mechanism of steel slag tailings after iron recycling under carbonation curing and the durability of the carbonated steel slag tailings compacts in seawater for use as an artificial reef. Similar to steel slag, the steel slag tailings after iron recycling could be consolidated after carbonation curing. However, some particular differences were observed; these are summarized as follows:

(1) The carbonation consolidation ability of steel slag tailings depends on its CaO content in the carbonation active minerals.
(2) In the process of carbonation curing, both carbonation and hydration occurred in the steel slag powder compacts, while only carbonation occurred in the steel slag mud compacts.
(3) The compressive strength of carbonated steel slag powder compacts increased due to the generation of various hydration products in the early stage of seawater curing. However, their compressive strength decreased due to the decomposition of the newly formed hydration products in the middle stage of seawater curing. Their compressive strength remained unchanged when the generation and decomposition of hydration products reached equilibrium in the late stage of seawater curing.
(4) The compressive strength of carbonated steel slag mud compacts decreased due to the decomposition of their hydration products in the early and middle stages of seawater curing. Their compressive strength increased due to the transformation of calcite to vaterite in the late stage of seawater curing.

## Figures and Tables

**Table 1:** The main oxides and minerals compositions of the steel slag powder (SSD) and steel slag mud (SSW) (wt%).

Oxides	SSW	SSD	Minerals	Chemical Formula	SSW	SSD
CaO	37.57	40.42	Larnite	Ca_2_SiO_4_	30.7	24.2
Fe_2_O_3_	27.75	24.48	Alite	Ca_3_SiO_5_	5.5	0.8
SiO_2_	16.35	14.13	Lime	CaO	3.5	0.7
MgO	6.95	4.51	Portlandite	Ca(OH)_2_	3.8	0.6
MnO	3.56	4.12	Clinoptilolite	C-S-H	4.4	5.9
Al_2_O_3_	3.21	7.02	Katoite	C-S-A-H	-	1.2
P_2_O_5_	2.69	1.86	Calcite	CaCO_3_	1.2	2.6
TiO_2_	0.84	1.04	Magnesite	MgCO_3_	4.4	7.2
SO_3_	0.30	0.56	Brownmillerite	Ca_2_(Fe,Al)O_5_	19	-
V_2_O_5_	0.27	0.26	Srebrodolskite	Ca_2_Fe_2_O_5_	-	20.6
Na_2_O	0.13	0.23	Mayenite	Ca_12_Al_14_ O_33_	-	7.2
Cr_2_O_3_	0.11	0.63	Tricalcium Dialuminate	Ca_3_Al_2_O_6_	-	6.9
K_2_O	0.08	0.36	Wuestite	(Fe, Mg, Mn)O	19.7	9.3
Cl	0.04	0.29	Magnesioferrite	(Mg, Fe)_2_ O_3_	7.9	10.1
Others	0.14	0.10	Quartz	SiO_2_	1	2.7
Total C	0.33	0.63				
LOI	1.03	1.63				

**Table 2:** The weight of compounds in each 1000 g of simulated seawater.

Compound	NaCl	MgCl_2_	Na_2_SO_4_	CaCl_2_	KCl	NaHCO_3_	KBr	Total
Weight (g)	23.497	4.981	3.917	1.102	0.664	0.192	0.096	34.449

**Table 3:** Weight loss values for each sample at different temperature intervals.

	50–240 °C C-S-H Gel Ettringite	240–500 °C Ca(OH)_2_ Monocarboaluminate	500–800 °C Carbonates	800–1000 °C	LOI
SSW	1.6	0.2	0.1	−0.5	99.0
SSD	0.9	0.5	0.5	−0.2	98.4
W-C24	1.7	0.7	6.8	0.0	90.8
D-C24	2.5	1.2	6.6	0.2	89.5
W-W28	1.5	1.4	6.6	0.5	89.9
D-W28	1.5	1.8	6.6	0.5	89.6
